# Validity of the “Rate‐a‐Plate” Method to Estimate Energy and Protein Intake in Acutely Ill, Hospitalized Patients

**DOI:** 10.1002/ncp.10389

**Published:** 2019-08-13

**Authors:** Ingeborg M. Dekker, Jacqueline A. E. Langius, Stephanie Stelten, Henrica C. W. de Vet, Hinke M. Kruizenga, Marian A. E. de van der Schueren

**Affiliations:** ^1^ Department of Nutrition and Dietetics Amsterdam UMC Vrije Universiteit Amsterdam Amsterdam the Netherlands; ^2^ Department of Nutrition and Dietetics Faculty of Health, Nutrition and Sport The Hague University of Applied Sciences The Hague the Netherlands; ^3^ Department of Epidemiology & Biostatistics Amsterdam UMC Vrije Universiteit Amsterdam Amsterdam the Netherlands; ^4^ Department of Epidemiology & Biostatistics Amsterdam UMC Vrije Universiteit Amsterdam Amsterdam Public Health Research Institute Amsterdam the Netherlands; ^5^ Dutch Malnutrition Steering Group Amsterdam the Netherlands; ^6^ Department of Nutrition and Health Faculty of Health and Social Studies HAN University of Applied Sciences Nijmegen the Netherlands

**Keywords:** adult, dietary intake monitoring tool, nutrition assessment, validity

## Abstract

**Background:**

Prevalence of malnutrition in hospitals has been reported around 20% and increases during hospitalization. The “Rate‐a‐Plate” method has been developed to monitor dietary intake and identify patients whose nutrition status deteriorates during hospitalization, but has not yet been validated. The objective was to study the validity and reliability of the method (phase 1) and redesign and revalidate a revised version (phase 2).

**Methods:**

Detailed food records provided a reference method. A priori difference of >20% in energy or protein between the reference and the “Rate‐a‐Plate” method was determined as clinically relevant. Intraclass correlation coefficients were used to determine the reliability.

**Results:**

In phase 1, 24 patients were included with a total 67 test days. In phase 2, 14 patients were included, 28 test days. In phase 1, the “Rate‐a‐Plate” method underestimated intake by 422 kcal (29%, ICC 0.349, 95% CI 304–541) and 5.7 g protein (10%, ICC 0.511, 95% CI 0.0–11.5). Underestimation was found in 65% and 23% for energy and protein intake, respectively. Underestimation was higher when patients had higher intake. In phase 2, underestimation was 109 kcal (7%, ICC 0.788, 95% CI −273 to 56) and 3.7 g protein (6%, ICC 0.905, 95% CI −8.4 to 1.0). In 32% and 21% of the cases, energy and protein intake were underestimated.

**Conclusion:**

The revised version of the “Rate‐a‐Plate” method is a valid method to monitor energy and protein intake of hospitalized patients and can be filled out by nutrition assistants. A larger validation study is required.

## Introduction

Malnutrition is an acute or chronic condition in which a deficiency or imbalance of energy, protein, and/or other nutrients leads to weight loss and/or measurable, adverse effects of body composition (decreased fat‐free mass).^1^ Malnutrition is an important factor affecting health and disease and is associated with delayed wound healing, decreased mental and physical functioning, and increased morbidity, mortality, and length of hospital stay.^2^, ^3^ The prevalence of malnutrition in hospitalized patients has been reported to be around 20% and is known to increase with age. Studies report progressive malnutrition during hospitalization, often due to a deficient nutrition intake.^2^, ^3^

As malnutrition is one of the main domains that may influence loss of functionality, it has become part of the “Safety Programme for Older Patients Admitted to the Hospital,” launched by the Dutch Government from 2008 to 2012. In this program, preemptive malnutrition screening and treatment interventions are advised to minimize the risk of (incident) malnutrition during hospitalization.^4^ Recording daily dietary intake of all older hospitalized patients, using the so‐called Rate‐a‐Plate method, has become part of this safety program in many Dutch hospitals, which is in line with the international European Society for Clinical Nutrition and Metabolism guidelines.^4^, ^5^ The Rate‐a‐Plate method is regarded as an easy method to obtain general estimation of dietary energy and protein intake and has been developed to recognize patients whose nutrition status may deteriorate during hospitalization because of insufficient dietary intake.^6^ The Rate‐a‐Plate method, filled in by nutrition assistants or nurses, classifies patients into 3 categories: low, moderate, and sufficient intake. Classified moderate intake requires in‐between meals, and with a low intake as a test result, referral to a dietitian is advised for a personalized intervention.^6^

Despite its widespread use in the Netherlands, the Rate‐a‐Plate method has not yet been validated. The objective of this study was therefore to determine the validity and interrater reliability of the Rate‐a‐Plate method and, if the method appears to be not valid, to redesign the Rate‐a‐Plate method into a valid version.

## Methods

### Study Design

**Figure 1:**
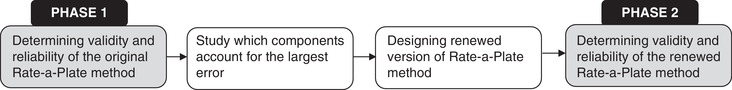
Flowchart of the design of the study.

The study was conducted at the Amsterdam UMC, Vrije Universiteit, the Netherlands, and consisted of 2 phases (Figure 1). In phase 1, the original Rate‐a‐Plate method was validated within the framework of a larger study called “Increasing Protein intake of Elderly” (VEvO‐study).^7^ Based on the findings, the Rate‐a‐Plate method was subsequently redesigned and revalidated (phase 2). The VEvO‐study was approved by the ethics committee of the Wageningen University with local approval by Amsterdam UMC, Vrije Universiteit, and participating patients provided written consent. The local ethics committee decided that the Medical Research Involving Human Subjects Act (WMO) did not apply to phase 2 of the study, so further ethical approval was not required for phase 2, and no written consent was obtained.

### Study Population

The study population consisted of older patients, aged 55 years and over, who admitted to the Department of Internal Medicine of Medicine at Amsterdam UMC, Vrije Universiteit, and were screened for inclusion by the researcher in charge (IMD, SS). Participants were excluded if they had decreased consciousness, impaired cognition, or dementia; were receiving tube feeding or parenteral nutrition; or were admitted for palliative treatment.

For each patient, the following baseline characteristics were collected: gender, age, height, weight, body mass index, and malnutrition score of the Short Nutritional Assessment Questionnaire (SNAQ).^8^

### Reference Method

Daily dietary intake of patients was registered during 3 (phase 1) or 2 (phase 2) consecutive days. Dietary intake at breakfast, lunch, in‐between meals, and drinks was registered using standardized portion sizes. Components of dinner were weighted on a calibrated digital scale to the nearest gram before consumption, with the uneaten food on the plate weighted afterward (phase 1) or estimated from the proportion of a meal eaten (recorded by photographs) based on earlier collected weighted portion sizes (phase 2) by both the researcher and nutrition assistant in charge. Total energy and protein intake was calculated with use of the Dutch Food Composition Database (NEVO) in both phases.^9^

### Rate‐a‐Plate Method

The Rate‐a‐Plate method, developed by the Dutch Malnutrition Steering Group (www.fightmalnutrition.eu), is a method to roughly monitor patients’ dietary intake and expresses dietary intake in points that correspond to energy and protein intake. The nutrition assistants or nurses calculate points. As defined per protocol, patients with a poor intake (≤4 points) for ≥2 consecutive days or moderate intake (5–7 points) for ≥4 days consecutive days are referred to a dietitian to prevent further decline in nutrition status.^6^

In phase 1, the original version of the Rate‐a‐Plate, 1 point equaled 200 kcal and 10 g protein. For the purpose of this study, researchers who were all dietitians or nutrition and dietetic students received training on the Rate‐a‐Plate method before the start of the study. Nutrition assistants had received training when the Rate‐a‐Plate method was introduced into the hospital, half a year before this validation study. Since discrepancy exists in background knowledge, differences in outcomes of researchers and nutrition assistants were expected. Therefore, results are shown separately. Each day, both the researcher and nutrition assistant in charge filled out the offered and eaten amount of food in the Rate‐a‐Plate method, independently of each other.

**Figure 2:**
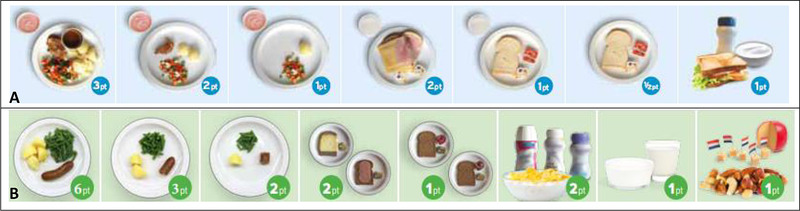
An example of the distribution of points according to (A) the original Rate‐a‐Plate method and (B) the renewed version of the Rate‐a‐Plate method.

Phase 2 studied which meals or which meal components accounted for the largest errors in phase 1. Most discrepancies were found for products highly contributing to protein intake (dairy products, oral nutritional supplements), a high intake of bread, or products rich in energy but not contributing to protein intake (lemonade or juices). In the adapted version, points were assigned for nonprotein foods and drinks by using a higher energy‐to‐protein ratio. An extra option for porridge or yogurt during breakfast and options for meat (one, half, or less than half) at dinner were added, and more points were allocated to oral nutritional supplements. In the revised version of the Rate‐a‐Plate (available at www.fightmalnutrition.eu), 1 point equals 130 kcal and 5 g protein. Figure 2A and 2B shows the examples of the distribution of points according to both versions of the Rate‐a‐Plate method. According to the manual of the Rate‐a‐Plate method, a score of ≤9, 10–15, and ≥16 points on 2 consecutive days is interpreted as low, moderate, and sufficient dietary intake, respectively.

### Statistical Analysis

Statistical analyses were performed using SPSS Statistics, version 20. Statistical significance was considered at the level of *P* < 0.05. To compare the Rate‐a‐Plate method with the reference method, total day score was calculated into energy and protein by multiplying the points by 200 kcal and 10 g protein (original version of Rate‐a‐Plate in phase 1) or 130 kcal and 5 g protein (revised version of Rate‐a‐Plate in phase 2). Only fully completed Rate‐a‐Plate forms were used for analyses. Paired sample *t*‐tests were used to determine differences between the reference method and the Rate‐a‐Plate method, and 95% CIs were calculated. Bland‐Altman plots were made for researchers and nutrition assistants separately to illustrate the mean intake and differences in intake based on the reference method and Rate‐a‐Plate method. On forehand, the research group decided by consensus that a difference of >20% between the 2 methods would be regarded as clinically relevant.

Intraclass correlation coefficients (ICCs) were used to determine the validity of the Rate‐a‐Plate method by comparing the Rate‐a‐Plate method filled out by both researchers and nutrition assistants with the reference method. A single 2‐way mixed model was used. An ICC > 0.75 was regarded as “good,” 0.40–0.75 as “fair,” and <0.40 as “poor.”^10^

The interrater reliability between the Rate‐a‐Plate method filled out by the researcher and nutrition assistant in charge was determined using ICC, with use of a single 2‐way random model.

## Results

### Study Population

Phase 1 consisted of 24 included patients. Data were completed for 1 patient for 1 day, for 3 patients for 2 days, and for 20 patients for 3 days of the study, which provided a total of 67 test days. Of the included patients, 33% were male, and mean age was 80.5 ± 10.3 years. At admission to the hospital, 42% of the patients had a SNAQ score ≥ 3, indicating malnutrition (Table 1).

**Table 1:** Characteristics of Included Patients

Characteristics	Phase 1	Phase 2
Gender, male^a^	8 (33.3%)	7 (50%)
Age, y^b^	80.5 ± 10.3	83.2 ± 10.1
Height, cm^b^	168 ± 11	171 ± 11
Weight, kg^b^	68.9 ± 14	69.7 ± 23.4
BMI, kg/m^2^ b	24.5 ± 4.4	23.9 ± 6.5
SNAQ score^a^		
0–2	13 (54.2%)	9 (64%)
3–7	10 (41.7%)	5 (36%)

BMI, body mass index; SNAQ, Short Nutrition Assessment Questionnaire.

a Data are displayed as n (%).

b Data are displayed as mean ± SD.

In phase 2, 14 patients were evaluated during 2 test days, which provided a total of 28 test days. Fifty percent of the patients were male, and mean age was 83.2 ± 10.1 years. According to the SNAQ score, 36% of the patients scored ≥3 (Table 1).

### Validity

#### Rate‐a‐Plate method filled out by nutrition assistants vs reference method

In phase 1, nutrition assistants filled out the Rate‐a‐Plate method during 40 study days. As presented in Table 2, the Rate‐a‐Plate method filled out by nutrition assistants underestimated the dietary intake by 422 kcal (difference with reference method 29%, *P *< 0.001) and 5.7 g protein (difference with reference method 10%, *P* = 0.100). In 27% and 68% of the cases, the Rate‐a‐Plate method accurately estimated the actual energy and protein intake. In 65% and 23% of the cases, energy and protein intake were underestimated. The ICC for the Rate‐a‐Plate method filled out by nutrition assistants, compared with the reference method, was 0.349 (95% CI 304–541) for energy and 0.511 for protein (95% CI 0.0–11.5).

**Table 2:** Dietary Intake by the Rate‐a‐Plate Method Filled Out by Nutrition Assistants Compared With the Reference Method (n = 40 Study Days in Phase 1 and n = 28 Study Days in Phase 2)

Dietary Intake	Reference Method^a^	Rate‐a‐Plate^a^	Differences^b^	95% CI
Energy, kcal				
Validation (phase 1)	1452 ± 415	1030 ± 364	−422 ± 371 (29.1%)	304–541
Revalidation (phase 2)	1599 ± 602	1490 ± 698	−109 ± 424 (6.8%)	−273 to 56
Protein, g				
Validation (phase 1)	57.2 ± 18.7	51.5 ± 18.2	−5.7 ± 17.9 (9.7%)	0.0–11.5
Revalidation (phase 2)	60.9 ± 28.6	57.2 ± 26.9	−3.7 ± 12.1 (6.1%)	−8.4 to 1.0

CI, 95% CI of the difference.

a Data are displayed as means ± SD

b Calculated as Rate‐a‐Plate minus reference method. Data are displayed as mean difference ± SD difference (%).

In the revalidation study (phase 2), data were complete in all 28 study days. The revised version of the Rate‐a‐Plate method filled out by nutrition assistants underestimated the dietary intake by an average of 109 kcal (difference with reference method 7%, *P* = 0.186) and 3.7 g of protein (difference with reference method 6%, *P* = 0.117; Table 2). The ICCs were 0.788 for energy (95% CI −273 to 56) and 0.905 for protein (95% CI −8.4 to 1.0). In 61% of the cases, the Rate‐a‐Plate method accurately estimated both energy and protein intake. In respectively 32% and 21% of the cases, energy and protein intake was underestimated.

**Figure 3:**
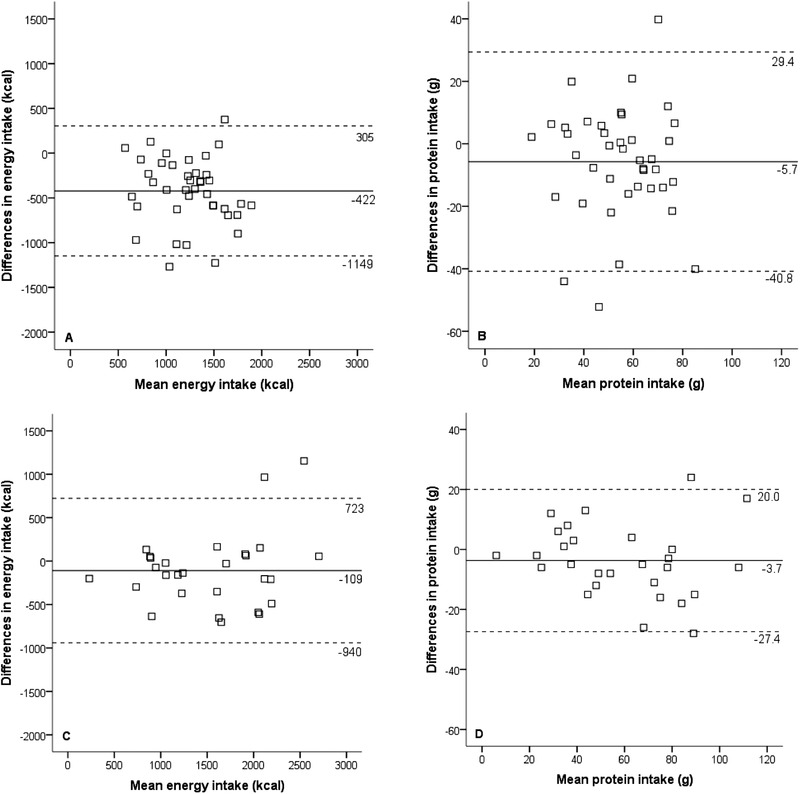
Bland‐Altman plot: Correlation of mean intake and differences in intake based on the Rate‐a‐Plate and reference methods filled out by nutrition assistants. Phase 1: (A) energy and (B) protein; phase 2: (C) energy and (D) protein.

Figure 3A–D illustrates Bland‐Altman plots of mean dietary intake and differences between the Rate‐a‐Plate method and reference method filled out by nutrition assistants.

Table 3 shows that the mean energy and protein intake over 2 consecutive days ascended stepwise from 863 ± 144 to 2092 ± 226 kcal and from 31 ± 12 to 88 ± 8 g protein for the low, moderate, and sufficient group of the Rate‐a‐Plate method.

**Table 3:** Mean Energy and Protein Intake Over 2 Consecutive Days Within the Low, Moderate, and Sufficient Group According to the Revised Rate‐a‐Plate Method (n = 28 Study Days)

Dietary intake	Low^a^	Moderate^a^	Sufficient^a^
Energy, kcal	863 ± 144	1626 ± 78	2092 ± 226
Protein, g	31.2 ± 12.4	53.1 ± 9.7	87.9 ± 7.9

a Data are displayed as means ± SD (range).

#### Rate‐A‐Plate method filled out by trained researchers vs reference method

In phase 1, researchers filled out the Rate‐a‐Plate method during all 67 study days. Mean difference between the reference method and the Rate‐a‐Plate method, filled out by trained researchers, was 515 kcal (35%, *P *< 0.001) and 9.7 g protein (17%, *P *< 0.001; Table 4). In 15% and 49% of the cases, the Rate‐a‐Plate method accurately estimated the actual energy and protein intake, respectively.

**Table 4:** Dietary Intake by the Rate‐a‐Plate Method Filled Out by Researchers Compared With the Reference Method (n = 67 Study Days in Phase 1 and n = 28 Study Days in Phase 2)

Dietary Intake	Reference Method^a^	Rate‐a‐Plate^a^	Differences^b^	95% CI
Energy, kcal				
Validation (phase 1)	1479 ± 471	964 ± 324	515 ± 379 (34.8%)	422–607
Revalidation (phase 2)	1599 ± 602	1562 ± 728	37 ± 402 (2.3%)	−193 to 119
Protein, g				
Validation (phase 1)	57.9 ± 19.2	48.2 ± 16.2	9.7 ± 15.4 (16.8%)	6.0–13.5
Revalidation (phase 2)	60.9 ± 28.6	60.0 ± 28.1	0.9 ± 7.9 (1.5%)	−4.0 to 2.2

CI, 95% CI of the difference.

a Data are displayed as mean ± SD.

b Calculated as Rate‐a‐Plate minus reference method. Data are displayed as mean difference ± SD difference (%).

**Figure 4:**
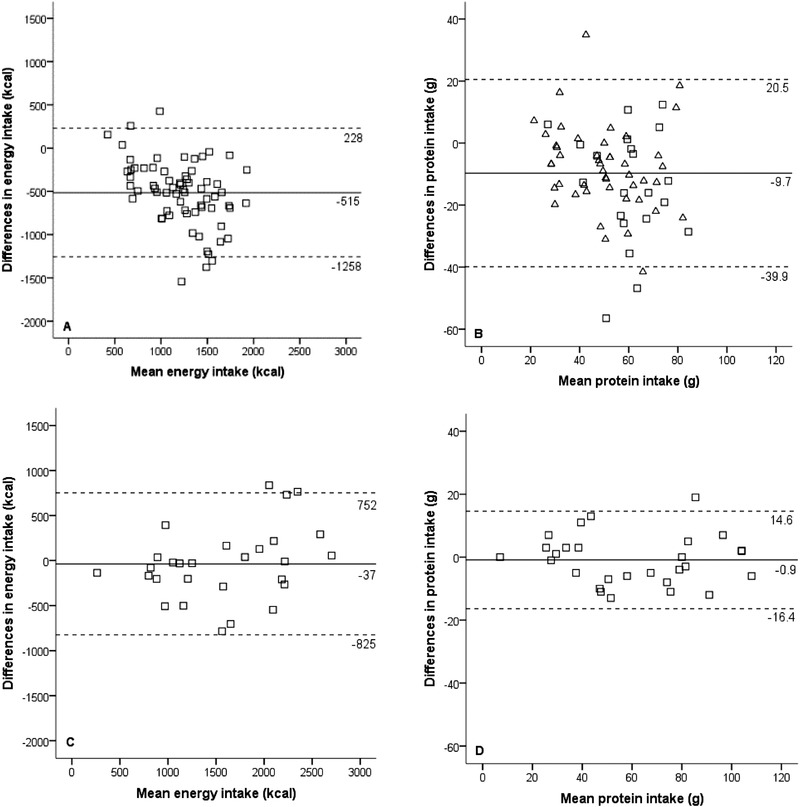
Bland‐Altman plot: Correlation of mean intake and differences in intake based on the Rate‐a‐Plate and reference methods filled out by researchers. Phase 1: (A) energy and (B) protein; phase 2: (C) energy and (D) protein.

In phase 2, researchers filled out the Rate‐a‐Plate method during all 28 study days. Mean difference between the reference method and the Rate‐a‐Plate method, filled out by trained researchers, was 37 kcal (2%, *P* = 0.633) and 0.9 g protein (2%, *P* = 0.541; Table 4). In 61% and 79% of the cases, the Rate‐a‐Plate method accurately estimated the actual energy and protein intake, respectively. Figure 4A–D illustrates Bland‐Altman plots of mean dietary intake and differences between Rate‐a‐Plate method and reference method filled out by trained researchers.

The ICC for the Rate‐a‐Plate method filled out by the researchers, compared with the reference method, was 0.311 (95% CI 422–607) for kcal and 0.545 (95% CI 6–13.5) for protein in phase 1 and 0.819 for kcal (95% CI −193 to 119) and 0.961 for protein in phase 2 (95% CI −4 to 2.2).

### Reliability

In phase 1, the ICC for interrater reliability between the Rate‐a‐Plate method comparing researchers and nutrition assistants was 0.441 (*P* = 0.003) for both energy and protein. In phase 2 of the study, this interrater ICC was 0.940 (*P *< 0.001) for energy and protein.

## Discussion

The objective of the present study was to determine the validity and interrater reliability of the Rate‐a‐Plate method, a method based on roughly monitoring patients’ dietary intake. The original Rate‐a‐Plate method underestimated mean energy intake by 30%, and this inaccurate estimated energy intake was correlated with increasing dietary intake. With this clinically relevant difference, a poor ICC, and an accurate estimated energy intake in fewer than one‐third of the patients, the original version of the Rate‐a‐Plate method was found to be invalid.^10^ Based on the findings, the Rate‐a‐Plate method was redesigned, and the validity and interrater reliability of this revised version were reanalyzed. The revised version of the Rate‐a‐Plate method had better energy and protein estimation, and twice as many patients had accurate energy estimates.

This is the first study assessing validity of a simple dietary intake monitoring tool for random daily intake in hospital inpatients. Other studies validated the estimation of separate food components by quoting in one‐half (0%, 50%, or 100%) and one‐quarter portions (0%, 25%, 50%, 75%, or 100%) or by estimating the total daily intake using a plate diagram based on a standardized daily menu.^11^, ^12^ Both methods therefore used a fixed energy and protein content of the products. The advantage of this method is that it is valid for monitoring ad libitum intake.

The differences between trained researchers and less‐well‐trained nutrition assistants suggest that nutrition education leads to more accurate data collection, most likely because professionals trained in nutrition have a better understanding of the energy and protein content of foods. Importantly, the revised Rate‐a‐Plate was found to be also valid when filled out by nutrition assistants, who are not professionals trained in nutrition. These results indicate the need for training prior to introducing the Rate‐a‐Plate, as training leads to better estimates, but the method has proven to be valid without training as well. The method could be built into an application to facilitate data collection by nutrition assistants.

The underestimation of energy in the original Rate‐a‐Plate raised with increasing energy intake. This was not the case for protein. An explanation could be that the original Rate‐a‐Plate focused mainly on products that contain protein, since protein is thought to play an important role in maintaining muscle mass during hospitalization. In a thorough evaluation of the data, discrepancies were primarily found in products rich in energy and low in protein. Dairy products, oral nutritional supplements, porridge, and a high intake of bread also influenced the validity of the Rate‐a‐Plate method. In the design of the revised version of the Rate‐a‐Plate method, this was adapted.

Results of the revised version of the Rate‐a‐Plate were promising, and the deviation from the true intake was rather small (7% for energy and 6% for protein). It performed within the 20% deviation, which was on forehand decided to be clinically relevant, and ICCs were good. Thus, the revised version was found to be a valid method to roughly monitor patients’ energy and protein intake.^10^

On forehand, it was decided by consensus that a difference of >20% between the reference method and the Rate‐a‐Plate method would be regarded as clinically relevant. Since the Rate‐a‐Plate method is based on roughly monitoring patients’ dietary intake, a higher accuracy of the method cannot be expected.

The Rate‐a‐Plate method was developed to recognize and treat patients for whom nutrition status becomes insufficient during hospitalization because of insufficient dietary intake.^6^ The Rate‐a‐Plate method classifies patients according to their scores into 3 categories, with a moderate intake requiring in‐between meals and a low intake requiring referral to a dietitian. Therefore, classification in the accurate category is of even higher importance than the exact (mis)classification of energy and protein. Classifying patients according to the Rate‐a‐Plate method as low, moderate, and sufficient dietary intake demonstrated clear differences in energy and protein intake between the groups.

A strength of the study is that in both phase 1 and phase 2 of the study, 1 of the researchers was present at the ward during serving and collecting all (in‐between) meals. Daily intake of patients was registered. Intake at breakfast, lunch, in‐between meals, and drinks was registered using standardized portion sizes. Components of dinner were weighed before consumption, with the uneaten food on the plate weighed afterward in phase 1 and estimated from the proportion of a meal eaten in phase 2 of the study. Estimation of the proportion of the warm meal is less secure than weighing, although earlier‐investigated portion sizes were used. However, weighing food in the second phase of the study was not the preferred method, because of logistic and hygienic aspects.

Another limitation is the relatively high amount of missing data of the Rate‐a‐Plate method, filled out by nutrition assistants in phase 1. The nutrition assistants filled out the Rate‐a‐Plate method for 40 patient days but also failed to do so for 27 days. As previously suggested, an application could possibly be helpful for nutrition assistants recording patients’ nutrition intakes. In phase 2, data were completed in all 28 study days, potentially because all patients (in contrast with study patients only) at the ward were included, and clear instructions could be given to collect intake data in every person. This suggests that measuring dietary intake by the Rate‐a‐Plate method in all hospitalized patients might be preferable to selecting vulnerable patients specifically.

In conclusion, the revised Rate‐a‐Plate method is a valid method to roughly monitor energy and protein intake of acutely ill, hospitalized patients. The method can be filled out by (trained) nutrition assistants, dietitians, or dietetic interns. A larger validation study and/or a study in other healthcare settings such as nursing homes or rehabilitation centers and other age categories is required.

## Statement of Authorship

I. M. Dekker, J. A. E. Langius, S. Stelten, H. M. Kruizenga, and M. A. E. de van der Schueren contributed to the conception and design of the research; I. M. Dekker, J. A. E. Langius, and S. Stelten contributed to the acquisition and analysis of the data; I. M. Dekker, J. A. E. Langius, H. C. W. de Vet, and M. A. E. de van der Schueren contributed to the interpretation of the data; and I. M. Dekker and J. A. E. Langius drafted the manuscript. All authors critically revised the manuscript, agree to be fully accountable for ensuring the integrity and accuracy of the work, and read and approved the final manuscript.
